# The reunion of two lineages of the Neotropical brown stink bug on soybean lands in the heart of Brazil

**DOI:** 10.1038/s41598-018-20187-6

**Published:** 2018-02-06

**Authors:** Patricia L. Soares, Erick M. G. Cordeiro, Frederico N. S. Santos, Celso Omoto, Alberto S. Correa

**Affiliations:** Department of Entomology and Acarology, University of Sao Paulo, Luiz de Queiroz College of Agriculture (USP/ESALQ), Piracicaba, SP 13418-900 Brazil

## Abstract

The rapid pace of conversion of natural areas to agricultural systems is highly concerning, and the consequences for conservation and pest management are not yet fully understood. We examined mitochondrial (COI and Cyt*b*) and nuclear (ITS1) gene regions of 21 populations of the stink bug *Euschistus heros*, to investigate the genetic diversity, genetic structure, and demographic history of this emerging soybean pest in South America. Two deep lineages that diverged in the Pliocene (4.5 Myr) occur over wide areas of Brazil. Historical changes during the Plio-Pleistocene led to significant genetic differences between *E*. *heros* populations, which differentiated further in several biomes. The northern lineage is older, more diverse, and prevalent in the Amazon and Caatinga, while the southern lineage is younger, less diverse, and prevalent in the Atlantic Forest and Chaco biomes. *Euschistus heros* populations are expanding in size and range but at different rates, strongly affected by environmental variables. Secondary contact between the main lineages is now occurring, mainly in areas of intensive farming and particularly in the Cerrado, an important agricultural frontier. Individuals adapted to different environmental conditions and to large monocultures might currently be combining into a panmictic and hard-to-control pest population.

## Introduction

The drastic climate changes during the Plio-Pleistocene have been considered the main cause of high levels of diversification in many areas in Brazil^1–3^. The four major Brazilian biomes, the Atlantic Forest (AF), Cerrado (central savannas), Amazon and Caatinga (seasonally dry tropical forest) have undergone profound changes during glaciation and interglacial cycles^4^. Due to this complexity and landscape composition, the processes of diversification by vicariance and habitat refugia are frequently invoked to explain the high levels of species endemism and diversity in this part of the planet^5–8^. Recent findings suggest that forest expansion and contraction and ‘historically stable areas’ may also have played a major role in the differentiation of lineages^9^.

Today, the Amazon and Atlantic forests are separated by a unique mosaic composed of savannas and woodlands that covers a large area between the two forest biomes, from Argentina and Paraguay (the Argentinean and Paraguayan Chaco) and continues through the central Brazilian Cerrado to the Caatinga in northeastern Brazil. This belt of mostly sparse dry vegetation is known as the ‘major South American disjunction’^3,10,11^ and forms a natural barrier preventing the movement of organisms between the northern Brazilian biomes and the Atlantic Forest^12–14^. Recent human impacts on these biomes, particularly the expansion of agricultural areas, have drastically changed the landscape and likely the connection between ecosystems, rearranging patterns that began to be formed millions of years ago.

The expansion of agricultural frontiers necessitates conversion of the native habitat to agriculture^15^. Fragments of native vegetation become embedded in a matrix of cropland and pasture that will eventually affect the species and ecosystem dynamics^16^. In some instances, habitat loss can limit the geographic range of endemic species, although certain species may thrive in their new surroundings. Cropland areas can provide exploitable resources, e.g. the expansion of soybean cultivation in Brazil in recent decades^17^. Soybean crops were formerly limited to southern Brazil (Atlantic Forest); in the early 1970s, advances in farming methods and new varieties allowed soybean farmers to expand into a new and important frontier, the Cerrado^18–20^.

Farming in the Cerrado has had both negative and positive impacts over the last 40 years. The expansion of soybean crops caused extensive environmental impacts such as fragmentation and the loss of natural areas, a matter of great concern for ecologists and conservationists^2,21,22^. On the other hand, Brazil is the second largest soybean producer after the USA, and soybeans account for an important share of its GDP^23^. For these reasons, it is important to understand both the impacts of soybean expansion on connecting natural populations, and the influx of insect pests from natural areas into soybean croplands.

The Neotropical brown stink bug, *Euschistus heros* (Fabr. 1798) (Hemiptera: Pentatomidae), is one of the most important pests of soybeans^24^. This Neotropical native is widespread in South America, living in markedly different environments including the Amazon Forest, Cerrado, Caatinga and Atlantic Forest^25^. The dispersal ability of *E*. *heros* is not well known, but is considered to be poor, perhaps because of its limited flight activity and diapause behavior^26,27^. This polyphagous pest feeds on members of Fabaceae, Solanaceae, Brassicaceae, Compositae, and Malvaceae, and often reaches high population densities on soybeans^28–31^. Rarely reported before the 1970s^24,32,33^, *E*. *heros* has since increased in abundance and is now found in all major soybean-producing regions^24^. Recently, this pest was recorded in Argentina^34^ and Paraguay^24^, raising concerns regarding a possible range expansion to other locations in South, Central and North America^24^.

Here we present a genealogy of mitochondrial and nuclear DNA sequences from *E*. *heros*. We addressed several questions regarding the genetic diversity, population structure, and demographic history of *E*. *heros* populations in Brazil and Paraguay, examining the potential role of past events in the differentiation of lineages, and the recent events (i.e. soybean expansion) promoting the admixture of ancient lineages. Our first objective was to determine the genetic distribution, studying population divergence and population structure. Second, we investigated the demographic history of *E*. *heros* in different biomes in South America. Finally, we used a modelling approach to explore how environmental variables and soybean expansion can explain the genetic pattern of current populations of *E*. *heros* in Brazil.

## Results

### Genealogical inferences

**Figure 1:**
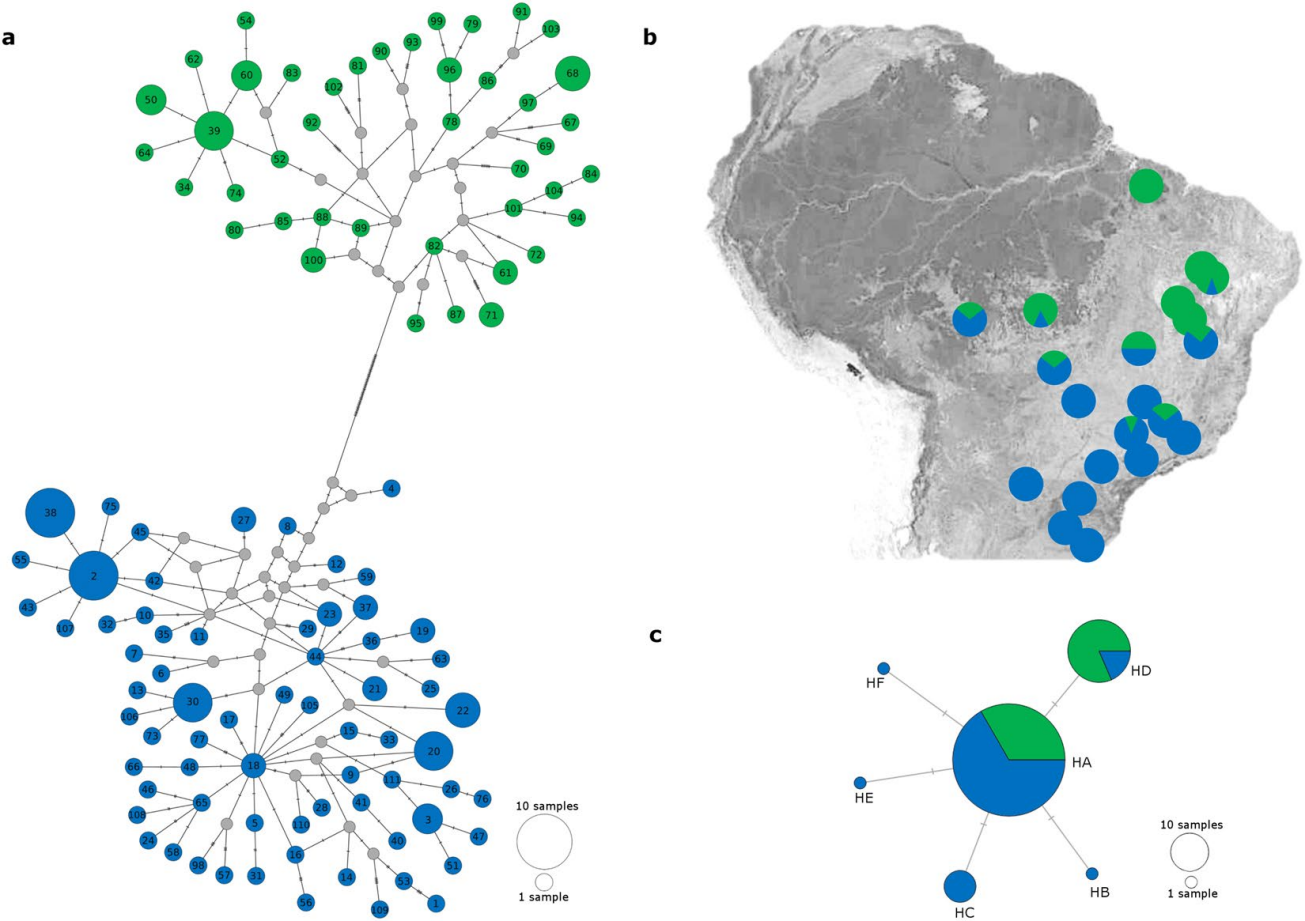
Median-joining network and geographic distribution of *Euschistus heros* haplotypes in South America. (**a**) Network of 159 concatenated mitochondrial COI-Cytb sequences. Size of haplotype circles reflects sample size, and gray nodes represent missing haplotypes. Colors indicate the two mitochondrial lineages of haplotypes: lineage N (northern) in green, and lineage S (southern) in blue. Number of mutation steps is shown as hatch marks. (**b**) Geographic distributions of the mitochondrial haplotypes. Circles represent the proportion of each lineage. The map was obtained from Google Maps, Map ©2017 Google, INEGI. Pie charts were drawn with Microsoft Excel. Maps were modified with GIMP 2.8.22 (**c**) Network of 124 nuclear ITS1 region sequences. The haplotype color refers to the mitochondrial lineages of the individual from which the ITS1 sequence was obtained.

Genealogical relationships among 111 mitochondrial haplotypes indicate two well-supported *E*. *heros* lineages separated by 52 mutational steps, and an estimated genetic distance of *D* = 0.042 (Fig. 1a). The southern lineage (S) haplotypes occur mainly in southern regions of South America (Fig. 1b). Small percentages of lineage S haplotypes were also found in central and northeastern Brazil (Fig. 1b), in a wide range of habitats (Table 1). The northern lineage (N) occurs mainly in northern and northeastern South America and was not present in Paraguay or southern Brazil (Table 1). A total of 91 haplotypes were identified as private haplotypes; the most frequent variants were H2 and H38 (*n* = 8), both from lineage S (Table 1).

**Table 1:** Sampling localities of *Euschistus heros*, with code, biomes, mitochondrial haplotype from two concatenated genes (COI-Cytb), haplotype nuclear ITS1 region, and geographic coordinates.

ID	Locations (City, State)	Code	Biome	mtDNA haplotypes (n)*	ITS1 haplotypes (n)	Latitude (S)	Longitude (W)
1	Teutônia, RS	RST	Atlantic Forest	*H1*; *H2(2)*; *H3*; *H4*; *H5*; *H6*; *H7*; *H8*	HA(4); HB	29°26′48.83″	51°48′50.44″
2	Santa Bárbara do Sul, RS	RSSB	Atlantic Forest	*H9*; *H10*; *H11*; *H12*; *H13*; *H14*; *H15*; *H16*; *H17*	HA(5)	28°22′01.95″	53°15′06.23″
3	Chopinzinho, PR	PRC	Atlantic Forest	*H18*; *H19(2)*; *H20(3)*; *H21*; *H22(4)*; *H23*; *H24*; *H25*	HA(6); HC(3)	25°51′23.28″	52°32′14.01″
4	Cornélio Procópio, PR	PRCP	Atlantic Forest	*H2(3)*; *H20*; *H26*; *H27*; *H28*; *H29*	HA(3)	23°10′57.89″	50°38′44.37″
5	Anhembi, SP	SPA	Atlantic Forest	*H23*; *H30(2)*; *H31*; *H32*; *H33*	HA(3)	22°47′17.09″	48°07′52.29″
6	Lavras, MG	MGL	Atlantic Forest	*H27*; *H43*; *H44*; *H45*	HA(2); HC	21°14′54.56″	45°00′04.95″
7	General Higinio Morínigo, PY	PY	Chaco	*H2*; *H20*; *H38*; *H105*; *H106*; *H107*; *H108*; *H109*; *H110*; *H111*	HA(3); HC(2)	25°09′19.55″	55°29′59.24″
8	Costa Rica, MS	MSCR	Cerrado	*H3*; *H30*; *H37*; *H48*; *H49*	HA(2); HC	18°32′37.15″	53°07′45.17″
9	Jaboticabal, SP	SPJ	Cerrado	*H2*; *H30*; **H34**; *H35*; *H36*; *H37*; *H38(2)*	HA(8)	21°15′09.05″	48°19′32.43″
10	Capitólio, MG	MGC	Cerrado	*H21*; *H30*; **H39(2)**; *H40*; *H41*; *H42*	HA(6); HD	20°36′50.88″	46°02′52.35″
11	Santa Juliana, MG	MGSJ	Cerrado	*H38(2)*; *H46*; *H47*	—	19°18′40.47″	47°31′57.69″
12	Padre Bernardo, GO	GOPB	Cerrado	*H18*; *H38*; **H39**; **H50**; *H51*; **H52**	HA(6); HD(3)	15°09′39.38″	48°17′01.46″
13	Rondonópolis, MT	MTR	Cerrado	**H39**; *H53*; **H54**; *H55*; *H56*; *H57*; *H58*	HA(7); HD; HE	16°27′55.71″	54°38′19.04″
14	Sorriso, MT	MTS	Amazon Forest	***H39***; **H50(2)**; *H59*; **H60**; **H61**; **H62**	HA(7); HD(2); HF	12°32′34.61″	55°43′17.53″
15	Cerejeiras, RO	ROC	Amazon Forest	*H2*; *H3*; **H60**; *H63*; **H64**; *H65*; *H66*	HA(5)	13°11′14.64″	60°49′02.48″
16	Paragominas, PA	PAP	Amazon Forest	**H67**; **H68(4)**; **H69**; **H70**; **H71(2)**; **H72**	HA(4); HD(4)	03°00′09.95″	47°21′11.19″
17	Correntina, BA	BAC	Caatinga	*H38(2)*; **H60**; *H73*; **H74**; *H75*; *H76*; *H77*	HA(8); HD(2)	13°20′33.19″	44°38′08.06″
18	São Desidério, BA	BASD	Caatinga	**H78**; **H79**; **H80**; **H81**; **H82**	HA(3); HD(5)	12°21′27.47″	44°58′38.23″
19	Luís Eduardo Magalhães, BA	BALE	Caatinga	**H61**; **H83**; **H84**; **H85**; **H86**; **H87**; **H88**; **H89**; **H90**	HA(3); HD(6)	12°05′25.63″	45°46′49.94″
20	Bom Jesus, PI	PIB	Caatinga	**H91**; **H92**; **H93**; **H94**; **H95**; **H96**; **H97**; *H98*; **H99**	—	09°04′17.95″	44°21′33.65″
21	Bom Jesus, PI	BJPI	Caatinga	**H96**; **H100(2)**; **H101**; **H102**; **H103**; **H104**	HA(2); HD(3)	09°04′17.95″	44°21′33.65″

*Italics haplotype = Southern lineage; Bold haplotype = Northern lineage.

Analyses of the ITS1 region revealed a single nucleotide polymorphism variation separating the haplotypes. The six haplotypes were separated by a one-step mutation (Fig. 1c and Supplementary material S2). We created two alternative ITS1 sequences for all individuals that showed ambiguity in the polymorphic site, to recreate the heterozygotes. The ITS1 network had lower haplotype diversity than the mitochondrial network. Haplotype HA was the most frequent (70.16%) and was widely distributed across all regions (Table 1). Haplotype HC was found in southern and central South America, and associated individuals previously identified as mitochondrial lineage S (Table 1 and Supplementary Fig S2). Haplotype HD (21.77%) was found only in northern South America, and associated individuals previously identified as mitochondrial lineages N or S (Table 1). The single haplotypes HB, HE and HF were found in populations RS1, MT1, and MT2, respectively (Table 1). The sharing of nuclear haplotypes by specimens from both mitochondrial lineages may indicate that these lineages can interbreed (Fig. 1c).

### Diversity statistics

These South American *E*. *heros* populations showed extensive mitochondrial diversity. Of 159 concatenated mitochondrial sequences of COI and Cytb analyzed, 111 haplotypes were found; most (82%) of these haplotypes were private and only 18% were shared among individuals. The overall haplotype diversity, nucleotide diversity and mean number of nucleotide differences were *h* = 0.991, π = 0.03312 and *K* = 32.892, respectively (Table 2). The haplotype diversity was similar in lineage N (*h* = 0.984) and lineage S (*h* = 0.982); however, lineage N (π = 0.0090) had higher nucleotide diversity than lineage S (π = 0.0062). For the different biomes, the haplotype diversity among biomes (groups) was low, ranging from 0.967 in the Amazon to 1.000 in the Chaco. The nucleotide diversity varied more widely among biomes, from 0.00627 in the Atlantic Forest to 0.03147 in the Amazon Forest (Table 2). Locations where both lineages were present had the highest levels of nucleotide diversity and mean numbers of nucleotide differences. Sequence analysis of the ITS1 region identified six haplotypes with a haplotype diversity of 0.461, nucleotide diversity of 0.0008 and mean number of nucleotide differences of 0.499 (Table 2). The haplotype diversity and nucleotide diversity were higher in lineage N (*h* = 0.503; π = 0.0008) than lineage S (*h* = 0.355; π = 0.0006), previously defined by the mitochondrial network. Among biomes, the highest diversity was observed in the Chaco (*h* = 0.600; π = 0.0009) and the lowest diversity in the Atlantic Forest (*h* = 0.315; π = 0.0005) (Table 2). The Amazon Forest, Caatinga and Cerrado have higher nucleotide diversity due to mixing of the two lineages in these areas.

**Table 2:** Measures of genetic diversity for *Euschistus heros* based on two concatenated mitochondrial genes (COI-Cytb) and ITS1 region.

Geographic regions	Sample size (*N*)	Haplotype number (*H*)	Haplotype diversity (*h*)	Nucleotide diversity (π)	Average # of nucleotide difference (*K*)
**COI-Cytb**					
Pooled	159	111	0.991	0.0331	32.892
Atlantic Forest	50	36	0.979	0.0063	6.224
Chaco	10	10	1.000	0.0066	6.511
Cerrado	37	27	0.970	0.0256	25.396
Amazon Forest	24	18	0.967	0.0315	31.246
Caatinga	38	35	0.996	0.0252	25.007
Lineage N*	57	42	0.984	0.0090	8.921
Lineage S*	102	69	0.982	0.0062	6.198
**ITS1 region**					
Pooled	124	6	0.461	0.0008	0.499
Atlantic Forest	28	3	0.315	0.0005	0.325
Chaco	5	2	0.600	0.0009	0.600
Cerrado	36	4	0.340	0.0006	0.357
Amazon Forest	23	3	0.466	0.0008	0.490
Caatinga	32	2	0.516	0.0008	0.516
Lineage N*	50	2	0.503	0.0008	0.503
Lineage S*	74	6	0.355	0.0006	0.382

*Lineages were previously defined by mitochondrial network.

### Mitochondrial divergence dating

**Figure 2:**
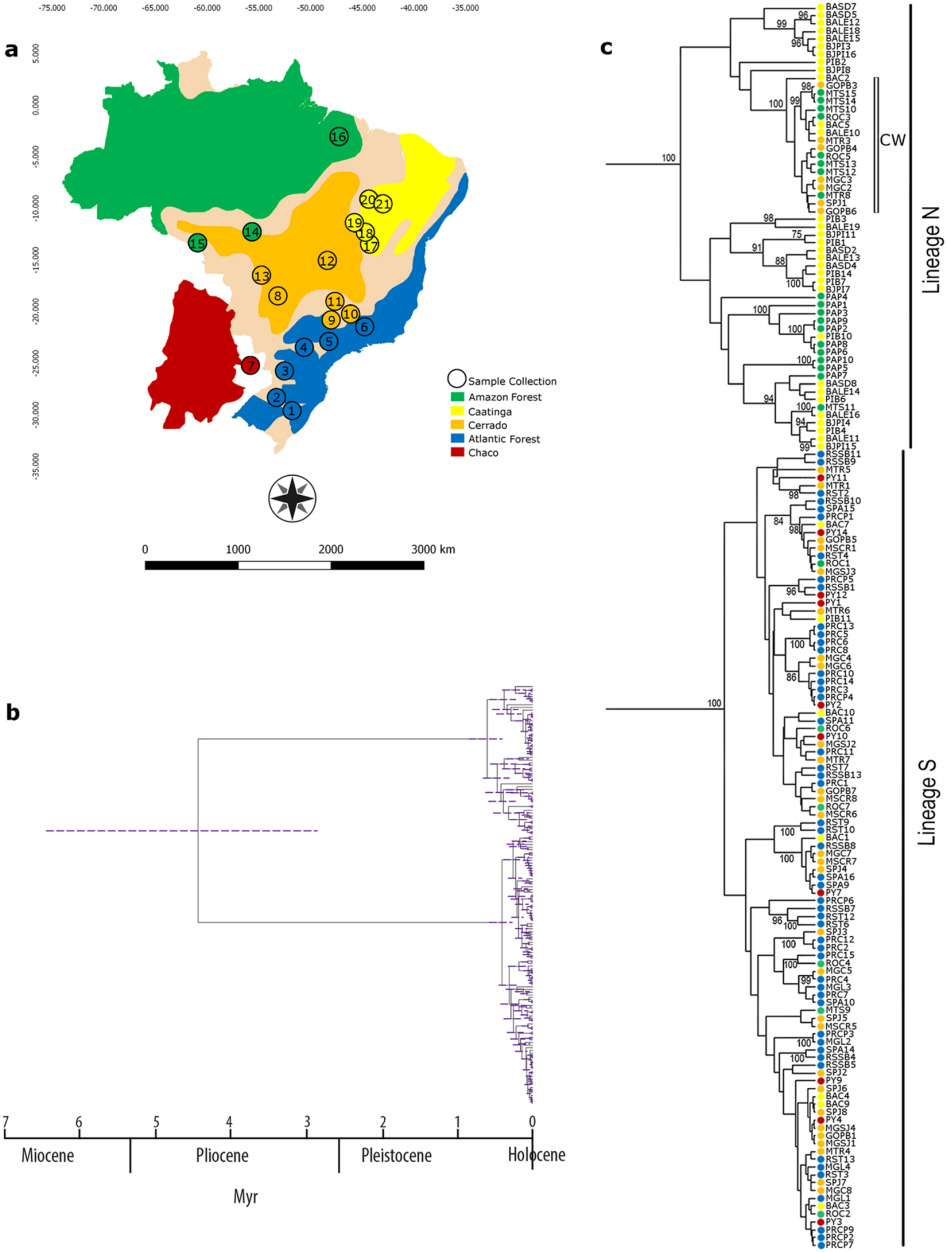
Bayesian coalescent tree for *Euschistus heros*. (**a**) Geographic and biome distribution of each population of *E*. *heros* sampled (see Table 1) (map based on Ab’Saber^77^ and modified with GIMP 2.8.22); (**b**) Bayesian phylogeny tree of 159 concatenated mitochondrial sequences (COI-Cytb). Gray bars at nodes indicate 95% highest probability density intervals (HPD) confidence intervals for nodal age. (**c**) Bayesian phylogeny tree showing posterior probability values (>75) and biome where individuals were collected (taxon names provided in Table 1).

The estimated age of origin of the lineage S (southern lineage) clade was 4.5 Myr (95% C.I 2.801–6.453 Myr), during the Pliocene, with intense diversification in the Pleistocene and Holocene (Fig. 2b).

### Population Structure

Lineage N haplotypes were associated mainly with the Amazon Forest and Caatinga, with one, more recent clade (CW) associated with central Brazil, a transitional region among the Cerrado, Caatinga and Amazon Forest (Fig. 2a,c). Lineage S predominates in the Atlantic Forest and Chaco, with a lower frequency in the Cerrado, Caatinga and Amazon Forest (Fig. 2a,c).

At the regional scale, the *E*. *heros* populations showed high genetic structure, as assessed by the Analysis of Molecular Variance (AMOVA). Differences among populations accounted for most of the genetic variances in mtDNA (56.57%, *P* < 0.001) and a high and significant value in ITS1 regions (20.18%, *P* < 0.001) (Table 3). A test of the hypothesis that the genetic variation is structured by biomes showed that 45.07% of the mtDNA total variance was distributed among biomes (Φ_CT_ = 0.450, *P* < 0.001). Furthermore, the larger portion of genetic variation within populations (39.61%, Φ_ST_ = 0.603) indicates overall genetic differentiation in these populations (Table 3). Analysis of the ITS1 region supported the mitochondrial data, showing a significant structuring by biome (Φ_CT_ = 0.173, *P* < 0.001), in which most of the genetic variation was within populations (Table 3).

**Table 3:** Analysis of molecular variance (AMOVA) for genetic structure of *Euschistus heros* based on two concatenated mitochondrial genes (COI-Cytb) and ITS1 region.

Source of variation	*d*.*f*.	Variance components	Percentage variance	Fixation indices (P-value)
(a) **COI-CytB**				
Among populations	20	9.550	56.57	Φ_ST_ = 0.566 (P = 0.00)
Within populations	138	7.333	43.43	
Total	158	16.883		
Among biomes	4	8.342	45.07	Φ_CT_ = 0.450 (*P* = 0.00)
Among populations within biomes	16	2.836	15.32	Φ_SC_ = 0.278 (*P* = 0.00)
Within populations	138	7.333	39.61	Φ_ST_ = 0.603 (*P* = 0.00)
Total	158	18.511		
(b) **ITS1 region**				
Among populations	18	0.050	20.18	Φ_ST_ = 0.201(*P* = 0.00)
Within populations	105	0.201	79.82	
Total	123	0.252		
Among biomes	4	0.045	17.30	Φ_CT_ = 0.173 (*P* = 0.00)
Among populations within biomes	14	0.014	5.49	Φ_SC_ = 0.066 (*P* = 0.07)
Within populations	105	0.201	77.21	Φ_ST_ = 0.227 (*P* = 0.00)
Total	123	0.260		

### Demographic statistics inferred for mitochondrial genes

Considering the two lineages, significant negative values were found in both the Tajima’s D and Fu’s Fs neutrality tests, indicating population expansion or purifying selection. Considering the biomes, the neutrality test statistics did not fully agree with one another. Fu’s Fs statistic was significantly negative for all biomes, but only the Atlantic Forest biome had a significant negative Tajima’s D value (Table 4).

**Table 4:** Neutrality test statistics and mismatch distribution analysis for *Euschistus heros* based on two concatenated mitochondrial genes (COI-Cytb).

Geographic regions	Sample size (*n*)	Tajima’s D	Fs de Fu	τ (SD_95%_)	SSD (*P*-value)	*r* (*P*-value)
**COI-Cytb**						
Pooled	159	0.445	−23.799*	56.2 (5.30 – 89.24)	0.0268 (P = 0.08)	0.0041 (P = 0.98)
Atlantic Forest	50	−1.920*	−25.224*	6.4 (4.25–7.48)	0.0016 (P = 0.66)	0.0136 (P = 0.55)
Chaco	10	−1.253	−4.883*	6.0 (2.81–7.01)	0.0325 (P = 0.12)	0.0563 (P = 0.34)
Cerrado	37	0.858	−18.632*	57.5 (3.66–79.53)	0.0394 (P = 0.09)	0.0160 (P = 0.80)
Amazon Forest	24	0.805	−7.198*	58.7 (3.98–196.99)	0.0363 (P = 0.10)	0.0419 (P = 0.26)
Caatinga	38	−0.067	−19.748*	7.3 (5.36–11.13)	0.0199 (P = 0.03*)	0.0071 (P = 0.84)
Lineage N	57	−1.779**	−24.741**	10.0 (7.25–11.03)	0.0112 (P = 0.04*)	0.0145 (P = 0.17)
Lineage S	102	−2.271**	−25.120**	6.5 (4.50–7.27)	0.0033 (P = 0.11)	0.0144 (P = 0.20)

Lineage were previously defined by mitochondrial network. τ = Expansion parameter; SSD = Sum of Squared Deviation; *r* = Harpending’s Raggedness Index.

For the lineages, the mismatch distribution analysis resulted in a nonsignificant SSD (*P* > 0.05), indicating a recent demographic expansion of lineage S but not lineage N (*P* = 0.04). For the biomes, a nonsignificant SSD (*P* > 0.05) was also found for the *E*. *heros* populations in all biomes but the Caatinga (*P* = 0.03) (Table 4). The nonsignificant raggedness index (*P* > 0.17) supports the spatial-expansion model of populations of lineages, biomes, and the entire group (all populations combined) (Table 4). The τ values were higher in the Cerrado and the Amazon Forest, τ = 57.5 and τ = 58.7, respectively, compared to the other three biomes, Atlantic Forest (τ = 6.4), Chaco (τ = 6.0) and Caatinga (τ = 7.3) (Table 4).

**Figure 3:**
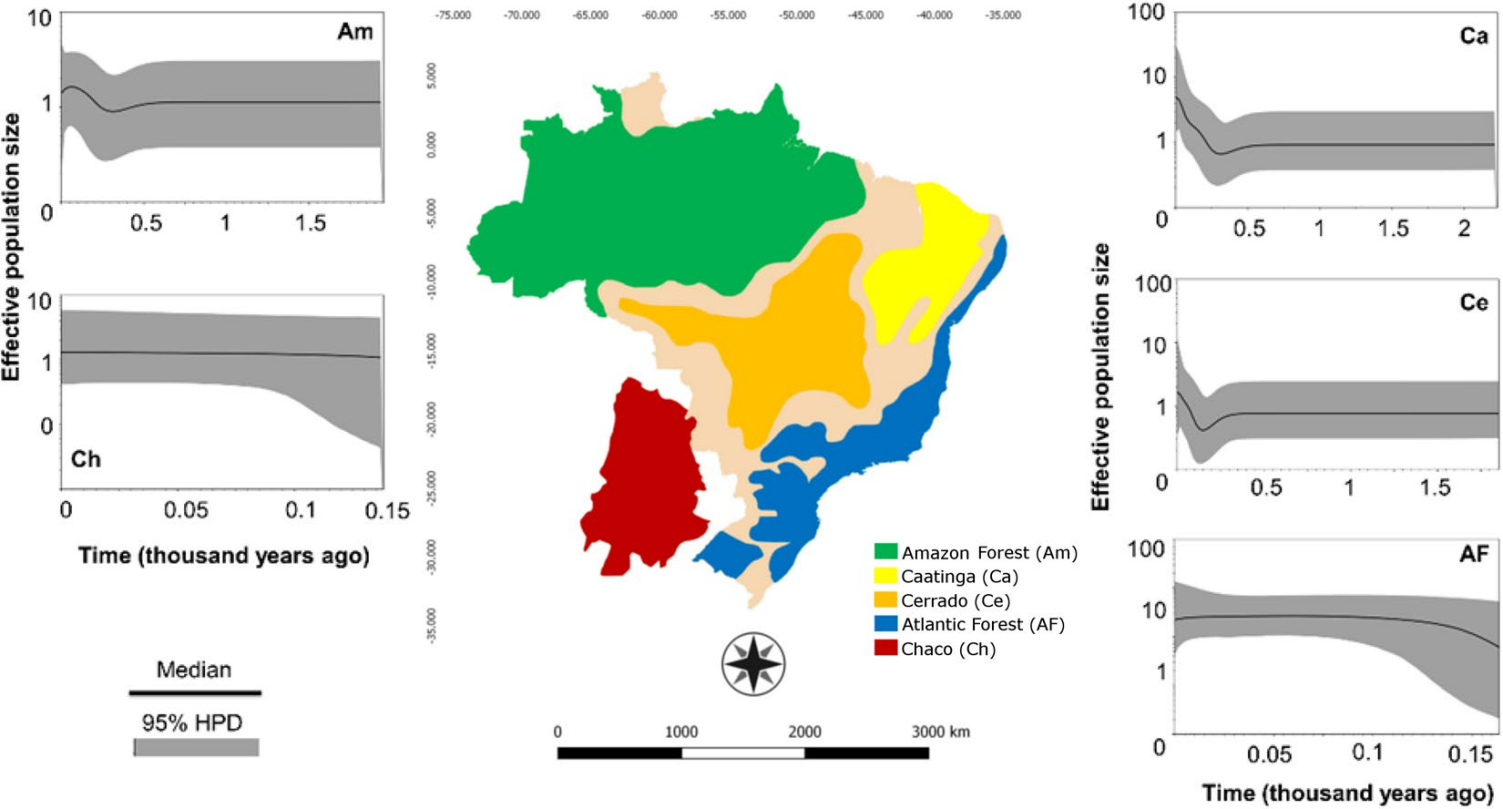
Bayesian skyline plot (BSP) showing population size dynamics for *Euschistus heros* in five biomes. The y-axis indicates effective population size (Ne) scaled by mutation rate (µ) as a function of time. Black horizontal line shows median BSP estimate, and gray area shows upper and lower 95% highest posterior density limits. The map was based on Ab’Saber^77^ and modified with GIMP 2.8.22.

The expansion of populations in the Amazon Forest, Chaco, Caatinga, Cerrado and Atlantic Forest occurred within the last 500 years, corresponding to a recent expansion during the Quaternary according to the Bayesian skyline plot analysis (Fig. 3). The Chaco and Atlantic Forest populations remained stable during the past 100 years, while the Caatinga and Cerrado populations are still expanding. According to the effective population size (Ne), the Atlantic Forest population is the largest, followed by the Caatinga. The Cerrado, Amazon Forest and Chaco populations have similar sizes, but the Cerrado population is still expanding very rapidly, while the Chaco population is expanding slowly and the Amazon population is now contracting (Fig. 3).

### Environmental features and soybean expansion modelling the current mitochondrial lineage distribution

**Figure 4:**
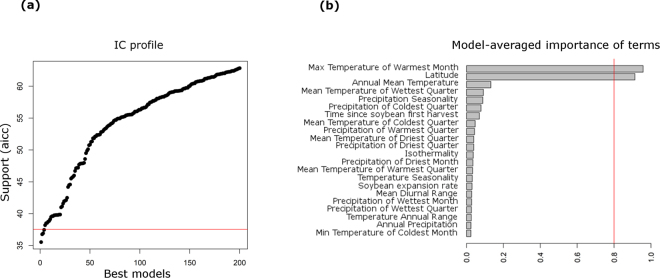
Model selection and variable importance. (**a**) AIC_c_ values for the 200 top models for percentage of the southern lineage. Horizontal red line separates models with AIC_c_ less than 2 units away from the “best” model. (**b**) Relative importance of predictors, considering all models. Relative importance is the sum of weights for the models in which the variable appears. Vertical red line indicates the 0.8 cutoff used to separate important variables.

Three models passed the cutoff (i.e. models that were less than 2 units away from the “best” model) to explain the presence (%) of the southern lineage at a given location (i.e. the probability of finding an individual from the southern lineage). The best predictors were the ‘max temperature of the warmest month’, ‘latitude’, and ‘annual mean temperature’ (Fig. 4 and Table 5). The most important variables were the ‘max temperature of the warmest month’ and ‘latitude’, which received the highest score in all top models. The model performance improved when ‘annual mean temperature’ was excluded, i.e. AIC_c_ (36.22 and 35.35) and *w*_*i*_ (0.09 and 0.14) (Table 5). The best model selected (AIC_c_ = 35.35) was 22.29 units away from the null model (AIC_c_ = 57.64). The variable ‘latitude’ (0.50) was more important than ‘max temperature of the warmest month’ (0.43) in the best model according to the *z-*scored beta (null deviance = 18 on 18 *d*.*f*., residual deviance = 4.04 on 16 *d*.*f*.) (Table 5). Latitude (0.9) was also the most important variable in the second-best model compared to the ‘max temperature of the warmest month’ (0.7) and ‘annual mean temperature’ (0.65) (null deviance 18 on 18 *d*.*f*., residual deviance = 3.469 on 15 *d*.*f*.) and in the third-best model (0.66) compared to ‘mean temperature of wettest quarter’ (0.35) and ‘max temperature of the warmest month’ (0.56) (null deviance 18 on 18 *d*.*f*., residual deviance = 3.531 on 15 *d*.*f*.). None of the three best models included soybean variables. The two soybean variables, ‘time since first soybean harvest’ and ‘soybean expansion rate’, ranked 7^th^ and 16^th^ in overall importance. The ‘time since first soybean harvest’ was strongly correlated with ‘latitude’ (*r* = 0.90, *d*.*f*. = 17, *P* = 0.000), ‘max temperature of the warmest month’ (*r* = 0.61, *d*.*f*. = 17, *P* = 0.005), and ‘annual mean temperature’ (*r* = 0.76, *d*.*f*. = 17, *P* = 0.000).

**Table 5:** Model-selection results showing the top three models for the response variable ‘presence of the southern lineage’.

Competing models		*AIC*_*c*_	*w*_*i*_ *(AIC)*
**Presence of southern lineage (%)**			
(1)	+0.50 Latitude −0.43 Max Temperature of Warmest Month	35.35	0.14
(2)	+0.90 Latitude −0.70 Max Temperature of Warmest Month +0.65 Annual Mean Temperature	36.22	0.09
(3)	+0.66 Latitude +0.35 Mean Temperature of Wettest Quarter −0.56 Max Temperature of Warmest Month	36.56	0.07

Selected models are those with lowest AICc values within two units from that of the best model. Best models are those with lower *AIC*_*c*_ values. The Akaike weights (*w*_*i*_) represent the relative likelihood of a model and can be used to compare the strength of evidence of alternative models.

## Discussion

Our results revealed two divergent deep lineages of *E*. *heros* in South America. The two COI-Cytb haplotype groups are separated by 52 mutational steps and have an estimated genetic distance of 4.2% (K2P). The number of mutation steps separating the two *E*. *heros* lineages is exceptionally high, raising the question of the possible presence of cryptic species. Rolston^35^ described a species in Middle America that is morphologically similar to *E*. *heros*, *Euschistus atrox* (Westwood) 1837, distributed in Colombia, Guiana, and Panama. However, examination of the external morphology, the sharing of ITS1 alleles by both lineages, and the admixture of lineages in the laboratory support the hypothesis of a single species that encompasses the two divergent lineages. Our results reveal the need for a thorough systematic review of the genus *Euschistus*.

The two *E*. *heros* lineages are geographically separated from one another, with one clade clustering the northernmost populations (i.e. northern and northeastern Brazil), and a second clade clustering the southernmost populations (i.e. southern and southeastern regions). Both mitochondrial lineages expanded to form a mixed zone upon secondary contact in the central and southwestern regions. It is not clear when the reunion occurred, but the formerly isolated populations seem to have come into contact before reproductive isolation was complete^36,37^. A related point to consider is that all but one of the northern haplotypes found in the Cerrado (CW) were phylogenetically grouped together in one clade, indicating a subgroup differentiation. The central-western (CW) subgroup likely occupied the region much earlier than the southern lineage arrived and before the first soybean fields were established. This is strong evidence against the hypothesis that the *E*. *heros* expansion was purely associated with the expansion of soybean cultivation during the 1970s.

The divergence time of the two main clades is estimated as occurring during the Pliocene (i.e. 4.5 Myr). This divergence seems to be associated with a cooling and drying of the global environment, which caused the separation of the Amazon Forest from the southern part of the Atlantic Forest and the consequent expansion of grasslands and savannas^38^. Temperature cycles were also associated with more recent diversification events during the Pleistocene (i.e. differentiation of the CW group). Deep sequence divergence dating to the Pliocene is also reported for other organisms in the Neotropics^39,40^, and phylogeographic structure has been found in amphibians in the Atlantic Forest^3^, reptiles in the Cerrado^41^, and plants^4,42^.

Spatial genetic structuring by biomes was also found among subpopulations of *E*. *heros*. Separation into the Amazon Forest, Caatinga, Cerrado, Atlantic Forest and Chaco biomes seems to be the best way to explain the genetic variance hierarchically. Thus, separating insects by biomes can help us to understand the pattern of lineage mixing, diversity and demographic history. The haplotype diversity of *E*. *heros* was high and similar among biomes and lineages. This pattern is the result of the high number of private haplotypes found in *E*. *heros* populations in all biomes. The higher nucleotide diversity of lineage N compared to lineage S can be explained under the ‘historical climate’ stability models, where a stable environment such as the Amazon Forest can offer conditions for a population to persist, resulting in elevated intraspecific genetic diversity^43,44^. Unstable regions, on the other hand, would be associated with recent or multiple-event colonization, resulting in lower intraspecific genetic diversity and signatures of expansion^4^. Therefore, the northern biomes (Amazon Forest and Caatinga) were the most stable environment, while the Atlantic Forest was the least stable environment. Another consideration is that lineage S is associated with areas that have undergone intense transformation due to agricultural practices, and has experienced population dynamics linked with farming cycles and control tactics.

Although *E*. *heros’* limited dispersal capacity likely helped to preserve the pattern formed during the late Tertiary and Quaternary as an outcome of the climate changes, the last 100 years were an important turning point for *E*. *heros* populations (i.e. soybean introduction and expansion of farming starting at the end of the 19^th^ century). It is plausible that farming and trade routes have increased the admixture process in certain areas, especially the Cerrado and connecting areas, even though there are still large areas where the two lineages have not yet encountered each other, showing that the pattern is still well preserved.

Recent signals of expansion were detected for *E*. *heros* lineages and in all biomes sampled. The inferences regarding population growth were supported by the neutrality tests, the unimodal mismatch distribution and the demographic expansion parameters (τ). Spatial expansion is also occurring, given that no significant Raggedness values were found. Apart from differences in test sensitivity, the lack of full agreement between tests for *E*. *heros* in each biome might indicate a more complex scenario. Multiple processes affecting local diversity and the noise from human intervention causing population reduction, population subdivision, bottlenecks, and facilitation of dispersal resulting in the secondary contact might affect the precise demographic estimates for a species^45–47^.

We also conducted a Bayesian Skyline analysis to test the hypothesis of recent expansion in all biomes and to determine how the effective sizes of the populations behaved over time. The period of *E*. *heros* population growth in all areas overlaps with the period of intense changes caused by the increase of urban occupation and agricultural area in South America^48^. It may be that the resulting habitat loss not only did not affect *E*. *heros* populations negatively, but has even been advantageous. One possible hypothesis to explain the success of *E*. *heros* is shifting hosts from natural areas to agricultural fields, especially soybeans but also cotton and bean fields^31^. A second hypothesis is that one or more traits occur in a latitudinal cline^49–51^. The species’ association with environmental gradients should also be considered, given the possibility of differences in traits and adaptations such as reproductive diapause^27,52,53^.

We used environmental and soybean variables to make phylogeographic inferences to predict the predominance of lineage S over lineage N at a given location. Selected models had similar AICc scores and considerably reduced the number of variables, down to 4 for the percentage of lineage S models. The two most important variables were the maximum temperature of the warmest month and the latitude. Temperature and photoperiod both affect this species, and might induce quiescence behavior and other possible differences in physiological responses. Latitude, on the other hand, can be correlated with geographic distance, environmental gradients, and agricultural gradients, as in the case of the soybean expansion. Our data support the predictions of the latitudinal-gradient hypothesis, even though distinct demographic scenarios can be expected at different times of *E*. *heros’* evolutionary history. The time since the first soybean harvest correlates with latitude and other bioclimate variables, which likely decreased the importance of this variable in the model.

The reunion of the two long-separated lineages might have unforeseen consequences for one of the largest soybean-producing regions in the world. The two lineages are united again in central Brazil, where an agricultural revolution started in the 1970s and continues today, pushing soybean fields northward^54^. It is possible that the northern and southern populations of *E*. *heros* are exchanging adaptations in admixture zones. However, knowledge of the differences between the two lineages is limited, because their presence was unknown until this point^55,56^. The changing status of *E*. *heros* from a secondary to a primary pest in soybean crops and the reasons for this are poorly understood. In recent years, the increase of population densities in soybean fields, the shorter quiescence period, larger host range (i.e. damage in cotton crops) and pesticide tolerance/resistance have been frequently reported in *E*. *heros* populations^30,31,57^. These concerns increase in a scenario of GM soybean introduction, no-till management, and expansion to diversity-hotspot areas.

## Material and Methods

### Sample collection and DNA extraction

One hundred fifty-nine specimens of *E*. *heros* were collected between 12/2015 and 07/2016 from 21 different localities across five South American biomes. Twenty sampling sites were in Brazil and one site in Paraguay. Specimens were collected as adults, from the canopy of soybeans, using a beating cloth under the plants. Individuals were preserved in ethanol (>95%) at –20 °C until laboratory manipulation, after which the remaining tissue from all specimens was stored at –80 °C. DNA was extracted from the head tissue of an adult specimen, using the modified CTAB protocol^58^.

### PCR amplification and DNA sequencing

Fragments of two mitochondrial and one nuclear region were amplified by polymerase chain reaction (PCR), using specific mitochondrial primers developed for this project and previously developed ITS1 primers^59^. The Cytochrome c Oxidase Subunit 1 (COI) fragment was amplified using the forward primer (5′-ACCGCACATGCATTTGTAATAA-3′) and the reverse primer (5′-GTGGCTGATGTGAAGTATGCTC-3′), and the Cytochrome b (Cytb) fragment was amplified using the forward primer (5′-GGATATGTTTTACCTTGAGGACA-3′) and the reverse primer (5′-GGAATTGATCGTAAGATTGCGTA-3′). To amplify the ITS1 rnDNA region (18 S partial – ITS1 complete – 5.8 S partial) we used the forward primer CAS18SF1 (5′- TACACACCGCCCGTCGTACTA-3′) and the reverse primer CAS5p8sB1d (5′- ATGTGCGTTCRAAATGTCGATGTTCA-3′). The PCR reactions were performed in a total volume of 25 μL containing 80 ng total DNA, 1.5 mM/μL MgCl_2_, 0.1 mM/μL dNTPs, 0.4 pmol/μL of each primer, 1 U of Taq DNA Polymerase (Synapse Inc.) and Buffer (10 × Taq DNA Buffer). PCR cycles consisted of denaturation at 95 °C for 3 min, followed by 35 cycles with denaturation at 95 °C for 30 s, annealing at 54 °C for 40 s, polymerization at 72 °C for 1.5 min and final extension at 72 °C for 10 min. Subsequently, the PCR products were separated on agarose gel (1.5% w/v) and observed under ultraviolet light. The amplicons were purified using 0.33 μL EXO I, 0.33 μL FastAp and 0.34 μL of ultra-pure water together with 10 μL of each PCR product, held at 37 °C for 30 min, then at 80 °C for 15 min. The PCR product Sanger sequencing was performed by the Animal Biotechnology Laboratory at ESALQ, University of São Paulo.

### Assembly of sequence datasets

All sequences were aligned and edited manually using the software Sequencher 4.0.1 (Gene Codes Corp., Ann Harbor, MI, USA). To eliminate missing data, sequences were interrupted at 607 bp for the COI gene, 386 bp for the Cytb gene and 638 bp (18 S partial – 52 bp; ITS1 complete – 416 bp; 5.8 S partial – 170 bp) for the ITS1 region. There were no insertions or deletions in the sequences obtained. All sequences (datasets) obtained in this study were deposited in NCBI-GenBank (https://www.ncbi.nlm.nih.gov/genbank/) with the accession numbers MG651970 - MG652128 (COI), MG652129 - MG652287 (Cytb), and MG654513 - MG654636 (ITS1).

The presence of nuclear paralogs of mitochondrial origin (termed numts)^60^ was inspected in the mitochondrial gene fragments, using the software MEGA v.5.2^61^. Three signatures of numts were searched: (*i*) indels that introduce frameshifts, (*ii*) out-of-place inframe stop codons that lead to premature termination of protein translation, and (*iii*) lack of codon position substitution bias toward the 3rd position, that lead to a higher rate of non-synonymous mutations. The presence of signatures (*i*) and (*ii*) is enough to consider a given sequence a numt. No numts were detected in the COI or CtyB sequences; therefore, we included all mitochondrial sequences in our analysis. The posterior analyses were performed using concatenated mitochondrial genes (COI-Cytb).

### Genealogical inferences

The genealogical relationships between haplotypes of the mitochondrial and ITS1 regions were reconstructed by a network of median-joining haplotypes, using the PopArt software^62^. Preliminary analysis revealed two putative mitochondrial lineages associated with *E*. *heros* populations in Brazil and Paraguay. The genetic distance (*D*) between the two mitochondrial lineages was inferred by dividing the haplotypes in two groups and calculating the 2-parameter Kimura method (K2P) in MEGA v.5.2 software^61^.

### Diversity statistics

The diversity analysis was performed by dividing individuals into two groups according to the mitochondrial lineages, or into five groups according to the biome to which the individuals belonged: Amazon Forest, Cerrado, Caatinga, Atlantic Forest or Chaco. Number of haplotypes, haplotype diversity (*h*), nucleotide diversity (π) and mean number of nucleotide differences (*S*) were estimated using the DNAsp v.5^63^.

### Divergence dating

We estimated the relative age of divergence between the two mitochondrial lineages using the Bayesian relaxed phylogenetic approach implemented in BEAST v.1.8.4^64^, based on the combined mitochondrial genes. The substitution model was determined using the software PARTITIONFINDER version 1.1.1.^65^ that selected the GTR + G + I model. A strict molecular-clock model to estimate the substitution rate and coalescent tree priors set to the constant size model were implemented. We used the insect molecular clock (mean = 0.0177, SD = 0.001)^66^ that corresponds to 3.54% pairwise divergence per Myr. Three independent runs were performed for 150 million generations, sampling every 1000 steps and discarding 20% as burn-in. TRACER v.1.6 was used to determine convergence, measure the effective sample size (ESS), and calculate the mean and 95% highest posterior density interval (HPD) for divergence times. Effective sample size (ESS) for all parameters exceeded 200, and the three runs converged to similar distributions. Runs were then combined with LogCombiner v.1.4.7^64^.

### Population Structure

Variance Analysis (AMOVA) was performed in Arlequin with parametric bootstrap (1000 replicates) using a 5% significance level^67^. The analyses were conducted to examine the presence of genetic structure among individuals, considering all sites sampled (non-hierarchical), among populations according to the sampling location (populations) and among biomes in three hierarchical levels.

### Demographic statistics inferred for mitochondrial data

Tajima’s D and Fu’s Fs neutrality tests were calculated using Arlequin v.3.5^67^. Both tests used 1,000 permutations using coalescing simulations. Fu’s Fs statistic was considered significant at the 95% confidence level when the *P*-value was less than 0.02. For each biome, we also estimated tau (τ) with its 95% confidence intervals, using a generalized least-squares approach and 1,000 coalescent simulations in Arlequin v.3.5. The parameter τ denotes the age of the expansion (*t*), so that *t* = τ/2 *u*; *u* = μ*L*g^68^. The parameter μ represents the estimated mutation rate, *L* is the length of the sequence, and g is the generation time. For *E*. *heros*, we did not estimate *t* directly, because the number of generations per year cannot be estimated straightforwardly. Thus, if we assume that the estimated mutation rate has not changed in *E*. *heros* (substitution rate = 1.345%), then *u* will be constant and we can consider that a smaller τ value indicates a newly established population, and a larger τ value an older one.

We conducted a mismatch distribution analysis using a spatial expansion model. The sum of square of deviations (SSD), raggedness index (*r*) statistics, and their associated P-value were calculated using Arlequin v.3.5. A nonsignificant SSD value means that the hypothesis of population expansion cannot be rejected, and a nonsignificant raggedness index indicates a good fit of the data to the spatial expansion model. We also used a Bayesian Skyline Plot (BSP) in Beast to reconstruct the demographic history, using TRACER v.1.6, based on the COI-Cytb data using 10 groups. We used the same substitution model and molecular-clock model that were used to estimate the divergence time.

### Environmental features and soybean expansion modelling the current mitochondrial lineage distribution

We used a model selection approach to identify and select variables that could be influencing the presence of a lineage at a given location^69,70^. Therefore, our response variable was the proportion of the southern lineage S calculated at each location as a percentage of the total composition. As predictor variables, we used ‘latitude’, 19 WorldClim variables based on all pixels of a CFR at 30 arc-second image^71^, and two soybean variables. The soybean variables consisted of the estimated time since the first harvest and the rate of increase of soybean production, given the cultivated area. We used linear regression to compile data from different sources and to estimate the two soybean variables, using the regression slope and the predicted year when the cultivated area was 100 hectares (Supplementary Table S1 and Fig. S1)^54,72,73^.

We evaluated the fit and plausibility of possible candidate models using glmulti^74^. We used a selection considering only the main effect, keeping the 200 best models. The criterion for selection was the corrected Akaike Information Criterion (AIC_c_)^75^. We selected models with AIC_c_ less than two units away from the best model. We also evaluated the Akaike weight of the best models, to assess the probability that a model is the best^76^. All variables were standardized by *z*-score, and the significance of each predictor was assessed by a GLM. We also assessed the importance of each variable by summing the Akaike weight for the models in which the variable appeared. Variables that appear many times in the top models, tend to be more important. We used the cutoff of 0.8 to separate the most important variables under the weight criterion^69,70^.

## Electronic supplementary material

Supplementary Information
