# Supplementary material for: The reunion of two lineages of the Neotropical brown stink bug on soybean lands in the heart of Brazil
Source: Sci Rep. 2018 Feb 6;8:2496. doi: 10.1038/s41598-018-20187-6 (PMC5802765; doi:10.1038/s41598-018-20187-6)

The reunion of two lineages of the Neotropical Brown stink bug in soybean lands in the heart of Brazil

Patricia L. Soares[1], Erick M. G. Cordeiro[1], Frederico N. S. Santos[1], Celso Omoto[1] and Alberto S. Correa[1]*

[1] Department of Entomology and Acarology, University of Sao Paulo, Luiz de Queiroz College of Agriculture (USP / ESALQ), Piracicaba, SP, 13418-900, Brazil

* Corresponding author:

Alberto Soares Correa
Department of Entomology and Acarology,
University of Sao Paulo, Luiz de Queiroz College of Agriculture (USP / ESALQ)
Av. Padua Dias, 11
Piracicaba, SP, 13418-900, Brazil
Tel. (+55) 19 3429-4199
Fax (+55) 19 3429-4120
E mail: ascorrea@usp.br

Supplementary Table S1 - **Summary of regression Analysis for soybean area expansion in Brazil**. Year as function of cultivated area.

Location	*β*	*Str.error*	*t-value*	*P*	*AdjR²*	*Predict year†*
MT	161.73*	10.22	15.82	P<0.001	0.83	1974
MS	40.18*	3.22	12.48	P<0.001	0.79	1963
GO	73.62*	2.95	24.92	P<0.001	0.93	1975
BA	27.21*	1.52	17.85	P<0.001	0.87	1973
PI	23.93*	2.21	10.82	P<0.001	0.82	1993
MG	25.87*	0.96	26.94	P<0.001	0.93	1969
SP	12.88*	1.07	11.98	P<0.001	0.73	1954
PR	92.37*	825	22.27	P<0.001	0.90	1963
RS	72.94*	6.14	11.87	P<0.001	0.71	1948
RO	7.26*	0.70	10.35	P<0.001	0.79	1991
PA	17.84*	2.65	6.72	P<0.001	0.71	2000

*P < 0.0001
†predict year when cultivated area was 0.1 thousand hectares (1000*ha)
Sources: Conab, IPEA, IBGE

Supplementary Fig. S1 – **Median-joining network and geographic distribution of *Euschistus heros* nuclear ITS1 haplotypes in South America.** (A) Network of 124 nuclear ITS1 region sequences. Size of haplotype circles reflects sample size and number of mutation steps is shown as hatch marks. (B) Geographic distributions of the ITS haplotypes. Circles represent the proportion of each haplotype. The map has been obtained from Google Maps, Map ©2017 Google, INEGI. Pie charts were drawn with Microsoft Excel. Maps were modified with GIMP 2.8.22.

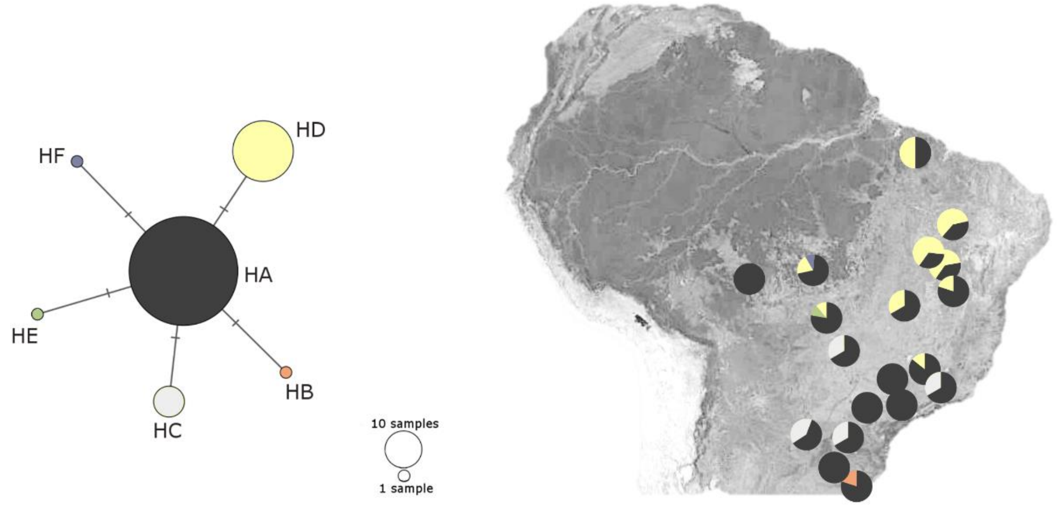

Supplementary Fig. S2 - **Soybean expansion rate.** Year as a function of soybean cultivated area (x 1000ha) in eleven states in Brazil.

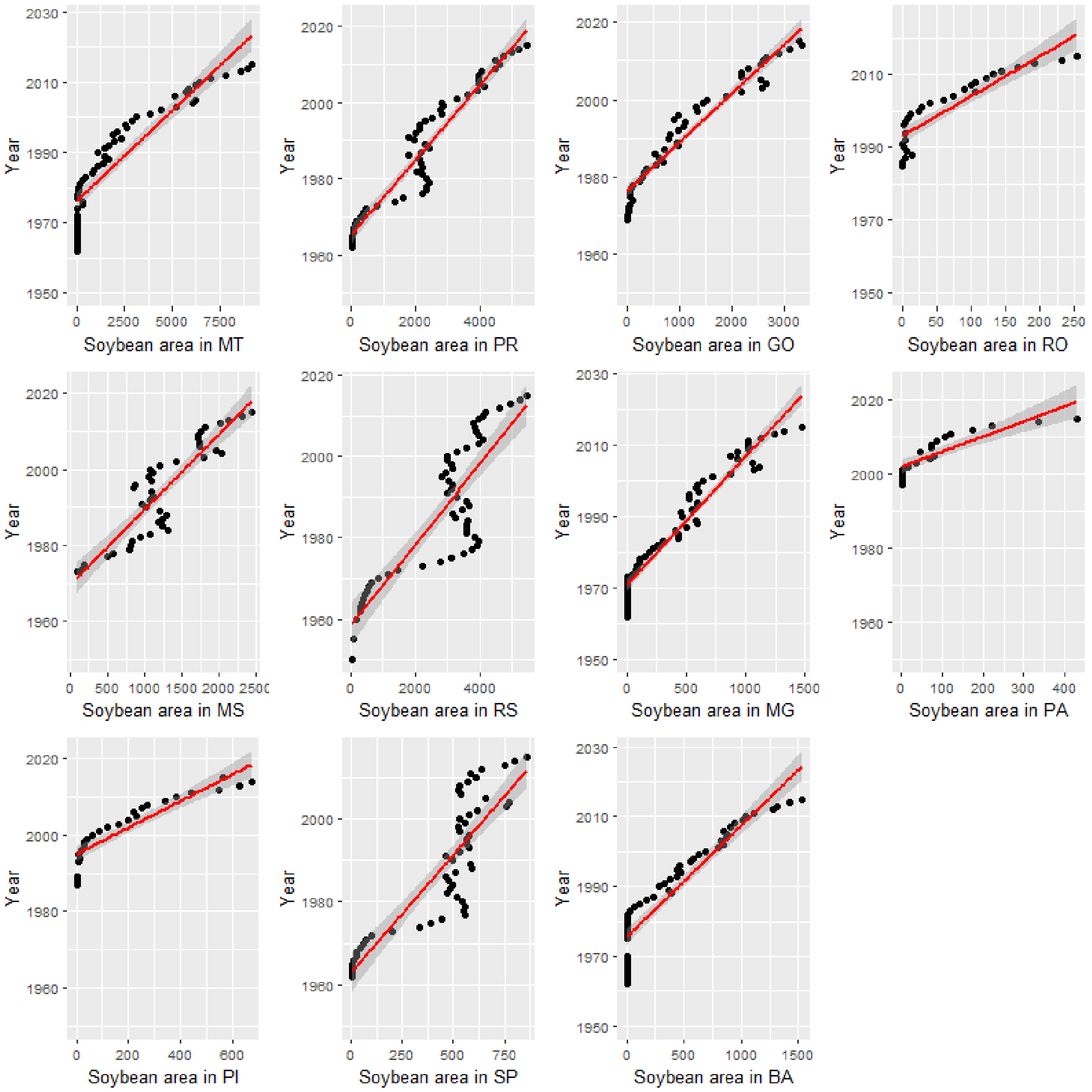

Supplement: Supplementary file 1 — Supplementary Information [file 41598_2018_20187_MOESM1_ESM.pdf]
